# Transport and Retention of Fecal Indicator Bacteria in Unsaturated Porous Media: Effect of Transient Water Flow

**DOI:** 10.1128/aem.00219-23

**Published:** 2023-07-17

**Authors:** Rozita Soltani Tehrani, Luc Hornstra, Jos van Dam, Dirk Gijsbert Cirkel

**Affiliations:** a Department of Soil Physics and Land Management, Wageningen University and Research, Wageningen, The Netherlands; b KWR Water Research Institute, Nieuwegein, The Netherlands; University of Delaware

**Keywords:** bacteria transport, unsaturated soil, transient flow, vadose zone

## Abstract

For production of clean drinking water, the processes governing bacterial remobilization in the unsaturated zone at transient water flow are critical. Although managed aquifer recharge is an effective way to dispose of pathogens, there are concerns about recontamination after heavy precipitation. To better understand how bacteria that were initially retained in porous media can be released to groundwater due to transient water content, transport experiments and modeling for Escherichia coli and Enterococcus moraviensis were conducted at the soil column scale. After inoculating dune sand columns with a bacteria suspension for 4 h, three rainfall events were performed at 24-h intervals. The effluent from sand columns was collected to analyze bacteria breakthrough curves (BTCs). After the rainfall experiments, the bacteria distribution in the sand column was determined. The collected BTCs and profile retentions were modeled with HYDRUS-1D, using different model concepts, including one-site kinetic attachment/detachment (M1), Langmuirian (M2), Langmuirian and blocking (M3), and two-site attachment/detachment (M4). After inoculation, almost 99% of the bacteria remained in the soil. The M1 and M2 bacteria models had a high agreement between observed and modeled concentrations, and attachment and detachment were two significant mechanisms for regulating bacteria movement in a porous medium with fluctuations in water flow. At the end of the experiment, the majority of bacteria were still found within the depth range of 5 cm to 15 cm. Our experiments show that E. coli is more mobile in sandy soils than E. moraviensis. The results of this study also suggest that the unsaturated zone is an important barrier between microbial contamination at the soil surface and groundwater. Follow-up studies are needed to completely understand the variables that regulate bacteria remobilization in the unsaturated zone of dune sands.

**IMPORTANCE** At managed artificial recharge sites in the Netherlands, recontamination of infiltrated water with fecal indicator bacteria has been observed. The results of this study suggest that the unsaturated zone is an important barrier between microbial contamination at the soil surface and groundwater. Bacteria that accumulate in the unsaturated zone, on the other hand, can multiply to such an extent that they can be released into the saturated zone when saturation increases due to major rain events or a rise in groundwater level.

## INTRODUCTION

Drinking water production in the western part of the Netherlands relies heavily on managed artificial recharge (MAR) of pretreated surface water in the coastal dune areas. Soil passage from infiltration basin to abstraction wells is an effective treatment stage in the production of drinking water, and its capacity to remove microorganisms is typically considered adequate (1–3). Providing adequate travel time through the porous medium is a cost-effective approach for reducing pathogen concentration in groundwater (4).

However, recontamination of infiltrated water with fecal indicator bacteria has been observed at MAR sites in the Netherlands. Fecal indicator bacteria are generally not harmful to humans but are indicators of fecal pollution, which might include harmful pathogens (5). These pathogens are thought to originate from feces deposited on the surface by grazing livestock introduced for nature management (cattle, sheep, horses) and wild fauna such as deer, foxes, and geese in the area between infiltration basins and abstraction wells. For instance, in the Castricum dune filtration area, enterococci have been found in water samples collected after infiltration and soil passage (6). Moreover, multiple disease outbreaks in the United States and Europe have been linked to polluted groundwater in recent decades (4, 7, 8). This suggests that the influx of bacteria from the surface may be larger than the straining and adsorption capacity of the vadose zone, particularly in terms of removing microorganisms from flowing water. While these associations suggest the potential contribution of bacteria influx from the surface, the adsorption capacity of the vadose zone alone may not fully explain the occurrence of drinking water-associated outbreaks. Factors such as geological characteristics, specific water pathways, and other site-specific considerations play important roles in determining the vulnerability of groundwater to contamination and subsequent disease outbreaks. Furthermore, certain bacteria can survive in the soil for up to 6 months (9) and can be remobilized and percolated to the groundwater by changes in soil moisture conditions (10).

**FIG 1:**
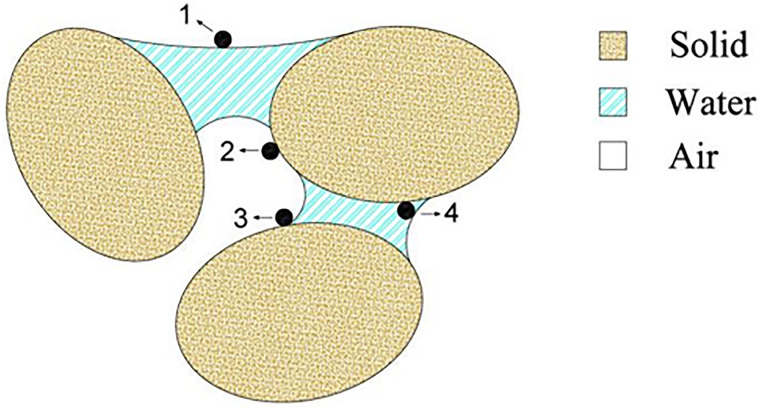
Schematic of colloid retention sites in unsaturated media. (1) AWI; (2) SAI (film straining); (3) SWA; (4) SWI.

The transport of colloids, such as bacteria and viruses, is influenced by the moisture level of porous media (11). Transient flow induces wetting and drying cycles in the vadose zone, causing abrupt changes in the local distribution of water and air, as well as capillary forces. Understanding mechanisms that drive microorganisms’ migration in the subsurface is critical for preserving water supply resources. Straining and adsorption are two mechanisms that contribute to the immobilization of colloids in porous media (12). When the drying front scours the soil grains during drainage, colloids can be resuspended in the receding water, but they can also be trapped inside thin water films that cover the soil grains that are left behind. Incoming water also introduces new colloids into the system, which can attach to soil grains at newly available sites vacated by the previous drying front. In unsaturated conditions, the presence of air as a third phase implies that air-water interactions exist. Figure 1 depicts a schematic of the various pore-scale colloid retention mechanisms. Bacteria and colloids have long been recognized to attach at air-water interfaces (AWIs) (13–17), implying that changes in soil air content influence bacterial movement. As retreating water films cover drained parts of the solid-water interface (SWI), the area of the AWI expands (15). In addition to electrostatic and van der Waals interactions from the SWI and AWI, a significant capillary force will act on retained colloids on the solid-air interface (SAI) if the water film thickness is lower than the colloid diameter (18). As water film thickness decreases, the colloids start to experience interaction energies from both the SWI and AWI, which will change the force and torque balance and may mobilize some colloids from the SWI to the aqueous phase or partition colloids from the SWI to the AWI (14).

Colloid attachment to the SWI in a saturated environment may come in the form of wedging or straining and bridging for numerous colloids (19–21). Mechanical filtration is used to retain colloids when every pore space in a porous medium is smaller than the diameter of colloids (22). Other comparable colloid retention mechanisms may occur in unsaturated conditions in addition to these saturated retention mechanisms. Colloid attachment can happen at the AWI (23). Colloid retention in thin water with a diameter less than the colloid diameter is known as film straining. At the solid, water, and air triple point (SWA), a colloid may also be preserved (24).

Recent research has shown that the dominant process controlling colloid transport, attachment, retention, and remobilization can vary significantly depending on the flow regime (e.g., steady state versus transient), as well as the water content of the porous media. Changes in water content by imbibition and drainage cycles influence the arrival time, peak concentration, and travel distance of colloids transported in porous media (10, 25–28).

The objective of this research is to determine whether bacteria, initially retained in the unsaturated zone in dune sands, are released due to changes in water content and hence increase the risk of contamination of groundwater utilized in drinking water production. The experimental setting was intended to mimic an actual field situation in which pathogenic microorganisms, mainly from animal excrement, are washed out and infiltrated into the soil by rainwater and then are carried to the groundwater through the unsaturated zone. The experiments consisted of a series of unsaturated column experiments with sand and bacteria collected from the aquifer recharge site. At the top of the columns, cycles of precipitation events were conducted, resulting in various water content levels, while we monitored the subsequent release of bacteria within the columns.

## RESULT AND DISCUSSION

### Flow and hydraulic properties of soil.

**FIG 2:**
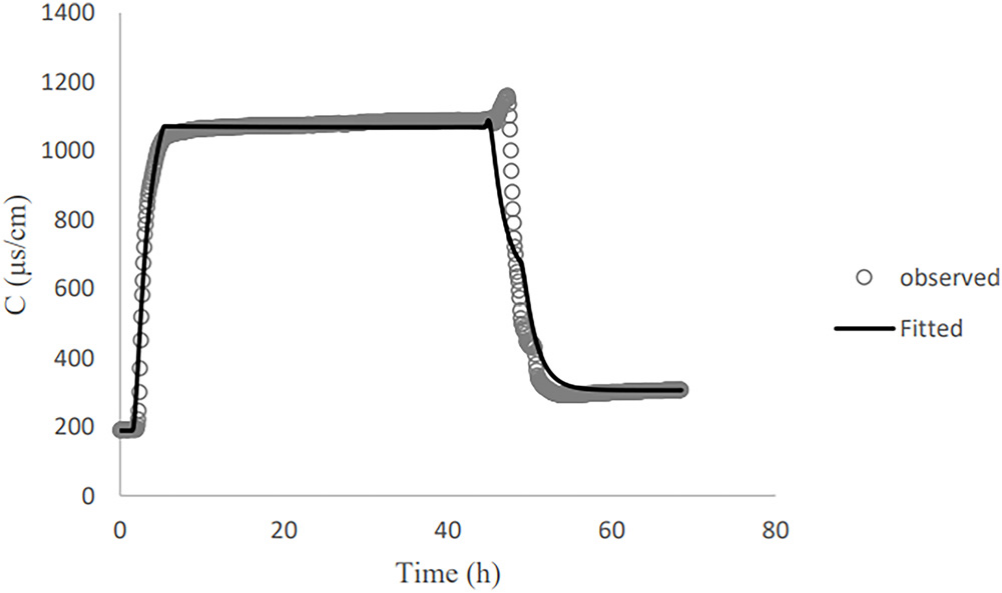
Observed and fitted NaCl breakthrough curve related to column 2.

Estimated hydraulic properties based on the soil moisture characteristic measurements (Table 1) are in line with measurements on undisturbed soil samples taken from the dune area. Optimizing the soil hydraulic conductivity (*K_s_*) and dispersivity using tracer transport data and inverse model in HYDRUS-1D also results in parameter values representative of dune soils. The convection-dispersion equation provides a good explanation of the NaCl breakthrough curves (BTCs) when used for the inverse optimization of the dispersivity (Fig. 2). Because of salt accumulation at the bottom of the column before the column leaches with clean water, there is a short surge at the end of the plateau (Fig. 2).

**TABLE 1:** Calibrated physical and hydraulic properties of sand columns^*a*^

ϴ*_r_*	ϴ*_s_*	α (cm^−1^)	*n* (−)	*K*_s_ (cm min^−1^)	*l* (−)	λ (cm)	ρ*_b_* (g cm^−3^)
0.001	0.47	0.01	2.26	3.85	0.5	0.26	1.51

a ϴ*_r_*, residual volumetric water content; ϴ*_s_*, saturated volumetric water content; α, parameter related to the inverse of the air entry value; n, pore size distribution index; *K_s_*, saturated hydraulic conductivity; *l*, tortuosity factor; λ, dispersivity; ρ*_b_*, bulk density.

### Bacteria mass balance.

The decay rate of both bacteria species was determined simultaneously with the column experiments, under the same condition, in two replications (equation 1 and Fig. S2-S3 in the Supplemental materials). The measured value in two replications was arithmetically averaged to calculate the die-off rate coefficients and the half-life time (Table 2). In comparison to Escherichia coli, Enterococcus moraviensis appeared to be more stable in unsaturated dune soil. According to studies, enterococci, to which E. moraviensis belongs, are present in the soil at large concentrations (29). Investigations revealed that enterococci have a stronger ability to survive than E. coli under environmental stress, which is consistent with our finding about the die-off rate of the bacteria (29–31).

**TABLE 2:** Measured bacterial decay rate

Species	μ^*a*^ (day^−1^)	Half-life time (days)
E. coli	0.29	2.4
E. moraviensis	0.08	8.3

a μ, decay constant.

The fitted decay rates were used to calculate the bacterial decay in the column outflow experiments. This allowed us to derive the mass balance, based on measured influent, effluent, final storage, and calculated bacterial decay (Table 3 and Tables S1-S4 in the Supplemental materials). The majority of the bacteria remained in the columns and did not percolate in the three rainfall events following the inoculation event. The mass balance calculation shows that in the case of E. coli, the total amount of percolated bacteria ranged from 0.003 to 0.06%. In the case of E. moraviensis, the total amount of percolated bacteria ranged from 0.0001 to 0.02%. The total mass balance error for the E. coli in the transport experiments ranged from −8 to +9%. However, the total mass balance error for E. moraviensis ranged from −32 to +31%. The observed higher error for E. moraviensis in the mass balance calculation can be attributed to various factors, such as the inherent challenges in accurately quantifying bacterial concentrations in soil and water samples, potential variations in decay rates, and the complex microbial processes that govern the transport and survival of E. moraviensis in unsaturated soil. These factors contribute to the overall uncertainty in the mass balance calculation and highlight the need for further investigation to better understand the underlying mechanisms.

**TABLE 3:** Experimental mass balance of E. coli and E. moraviensis for column 2

Mass type	Data for:			
	E. coli		E. moraviensis	
	CFU	%	CFU	%
Influent	2.10E+10	100	1.28E+08	100
Effluent	9.97E+05	0.005	8.74E+03	0.007
First rain	2.75E+06	0.013	9.73E+01	0.00007
Second rain	1.98E+06	0.009	1.73E+02	0.00013
Third rain	4.66E+05	0.002	8.60E+01	0.00007
Remain in the column	8.14E+08	4	7.10E+07	56
Decay rate	1.82E+10	87	5.77E+07	45
Error	1.94E+09	9	−1.03E+06	−0.80

### Bacteria transport modeling.

**FIG 3:**
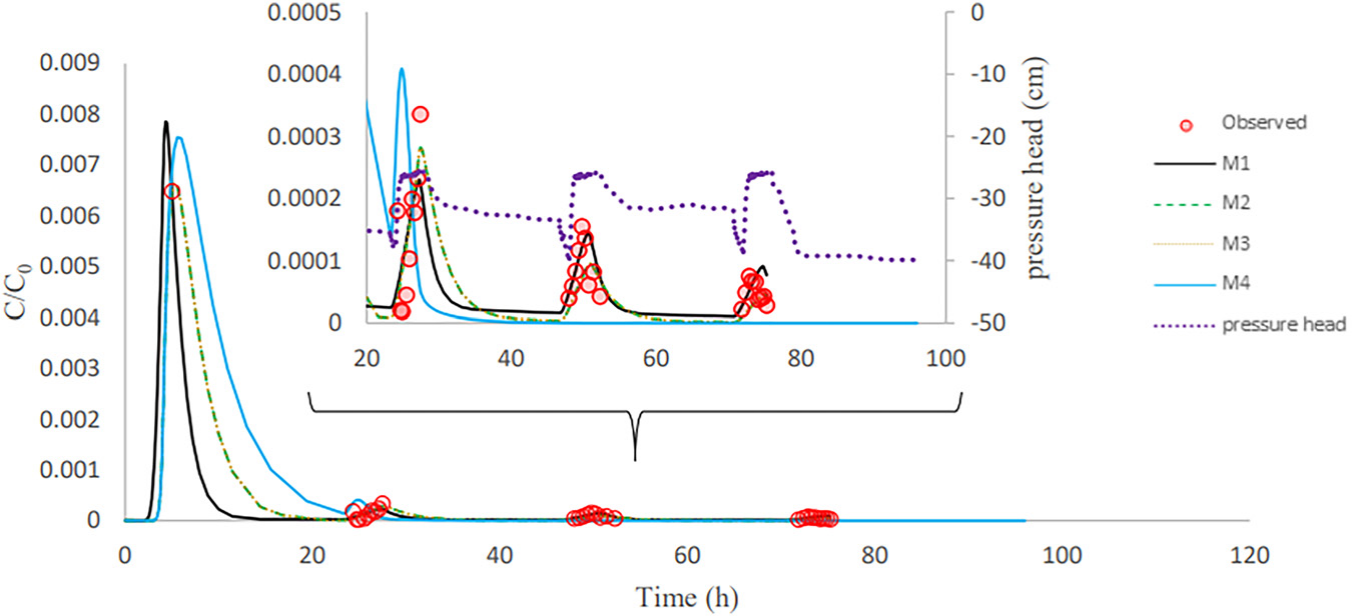
Observed and simulated breakthrough curves for E. coli in column experiment 2.

**FIG 4:**
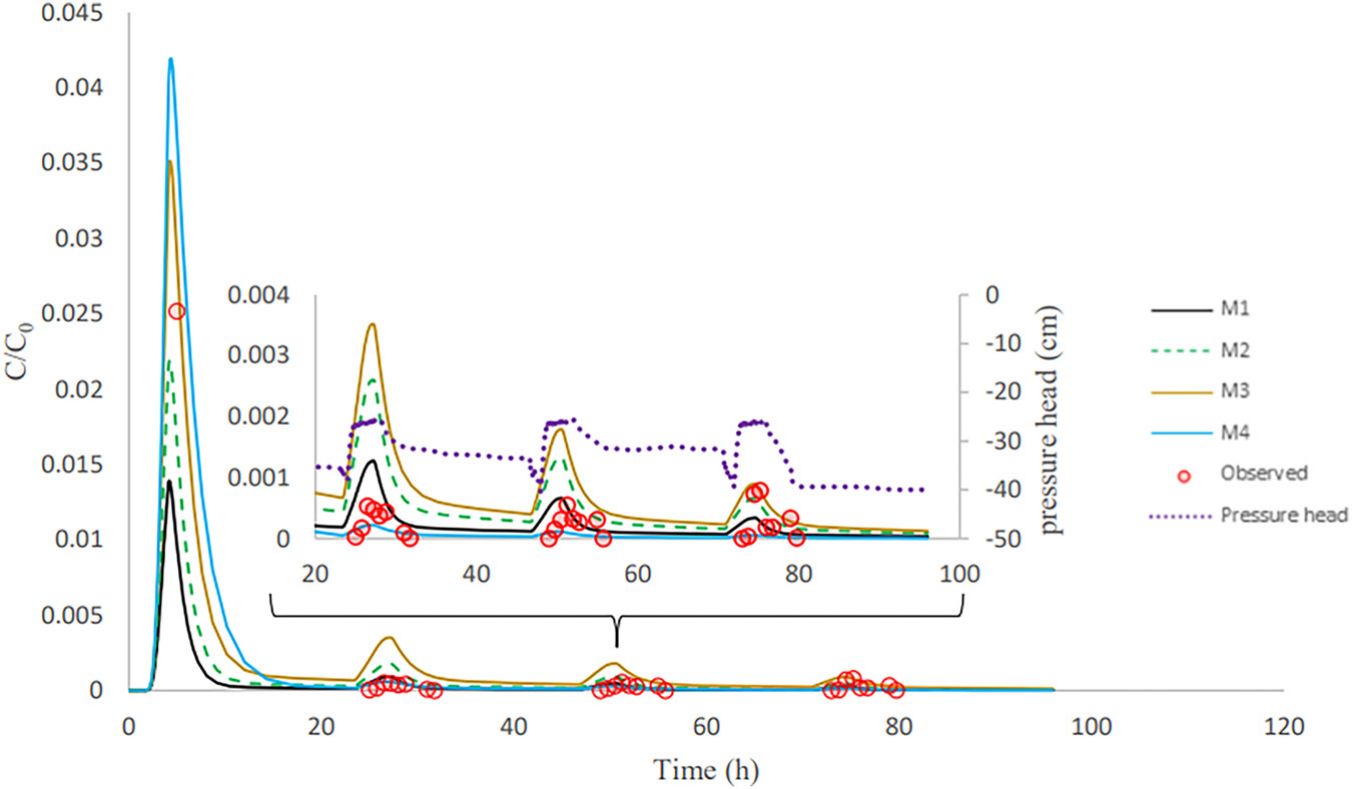
Validation showing observed and simulated breakthrough curves for E. coli in column experiment 5.

**FIG 5:**
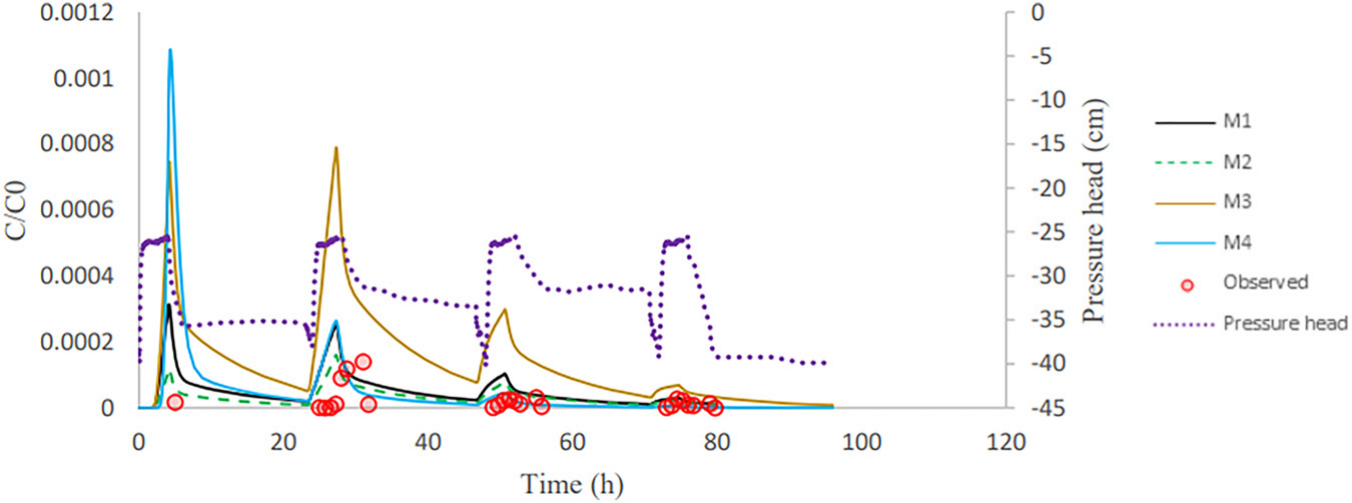
Calibration showing observed and simulated breakthrough curves for E. moraviensis in column experiment 4.

**FIG 6:**
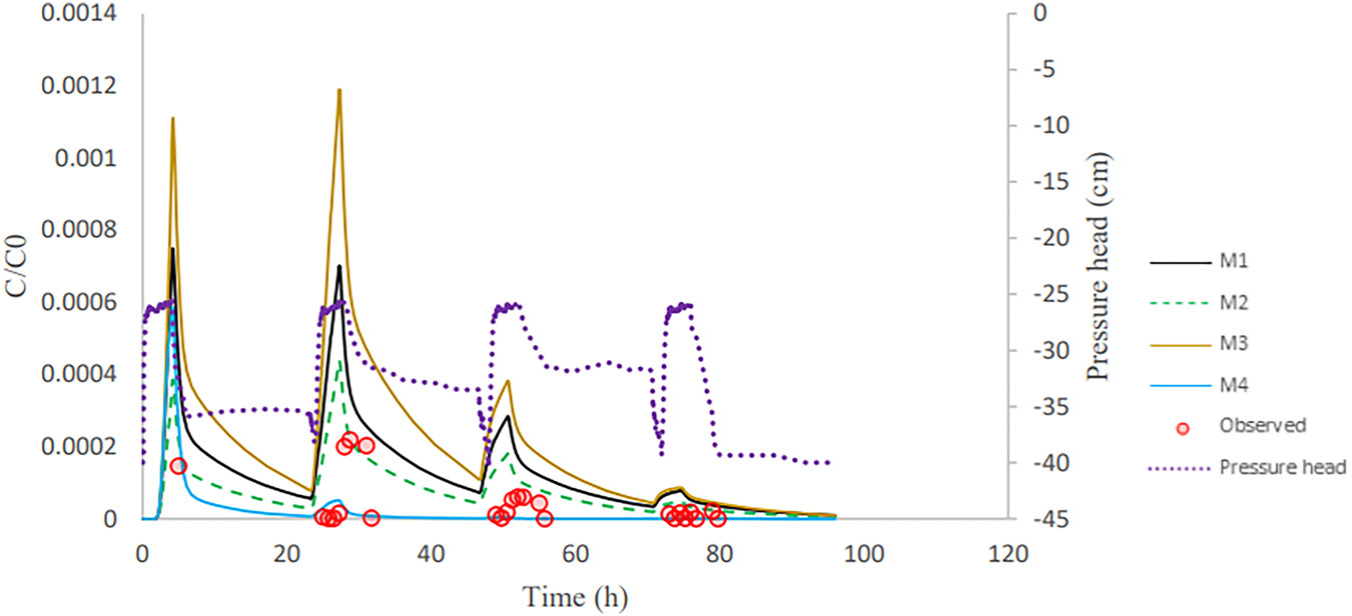
Validation showing observed and simulated breakthrough curves for E. moraviensis in column experiment 5.

After column inoculation, three rainfall events with tap water were applied to the column. This causes a high initial peak (inoculation effluent) and three subsequent bacteria peaks in the outflow (Fig. 3 to 6). The breakthrough curves illustrate variations in the relative bacteria concentration with respect to influent concentration after each rainfall event. The majority of bacteria in the soil column were attached to soil particles and interfaces before detaching from retention sites (Table 3). In all columns, the majority of the E. coli were released during inoculation, and the number of bacteria released reduced dramatically during subsequent rainfall events. E. moraviensis release, on the other hand, was not significantly higher during inoculation than during rainfall events, indicating that E. moraviensis adhered to soil particles and interfaces as soon as it entered the sand columns.

The lines in Fig. 3 to 6 illustrate simulated BTCs for E. coli and E. moraviensis transport for column experiments 2, 4, and 5. Bacteria were transported with the water flow in porous media. They collided with solid particles and interfaces due to convection and diffusion and could be subject to attachment. Column experiment 2 was used for model calibration, and column experiment 5 was for model validation. Simulations used the one-site attachment/detachment (M1), Langmuirian (M2), Langmuirian with blocking (M3), and two site depth-dependent (M4) models. Table 4 shows a summary of the model parameters that have been fitted, as well as statistical data on the goodness of fit. The Akaike information criterion (AIC) was used to evaluate the relative suitability of the four model formulations to describe the BTC data (32). It is desirable to use the model with the lowest AIC value. The simulated breakthrough curves from one-site model and Langmuirian model match the observed data reasonably well for E. coli (*R*^2^ > 0.9). It is clear from Fig. 5 that the Langmuirian model could simulate the observed breakthrough curve for E. moraviensis transport reasonably well (*R*^2^ > 0.6), and the performance of the three other models was rather poor. E. coli and E. moraviensis BTCs demonstrated greater agreement with the one-site attachment and detachment model and the Langmuirian model, respectively. This is supported by the model selection criteria (AIC and *R*^2^), which indicate that the performance of the one-site attachment and detachment model is better than other models for E. coli, and performance of the Langmuirian model is better than other models for E. moraviensis. It is clear from Fig. 3 to 6 and Table 4 that neither the Langmuirian with blocking nor the two-site depth-dependent model could satisfactorily simulate the breakthrough curve of E. coli and E. moraviensis. The above findings are in line with the values of AIC and *R*^2^. Similar to Gargiulo et al. and Bradford et al., we assumed this is because the majority of bacteria remained near the column entrance, and just a very small percentage moved to deeper levels in the soil (33, 34).

**TABLE 4:** Fitted model parameters for E. coli and E. moraviensis based on 5 columns and using 4 model formulations^*a*^

Bacteria	Model	*R* ^2^	AIC	Mean *K*_att2_ (min^−1^)	Mean *K*_det2_ (min^−1^)	Mean *S*_max_ (g^−1^ sand)	Mean *K*_att1_ (min^−1^)	Mean *K*_det1_ (min^−1^)
E. coli	M1	0.98	−147				0.80 (0.25)	0.01 (0.01)
E. coli	M2	0.98	−113			219.33 (379.83)	0.67 (0.24)	0.01 (0.01)
E. coli	M3	0.34	−93			1,656.39 (2,341.67)	0.54 (0.20)	0.01 (0.01)
E. coli	M4	0.07	−94	0.44 (0.26)	0.003 (0.002)		0.41 (0.71)	0.00004 (0.00007)
E. moraviensis	M1	0.55	−358				2.25 (0.52)	0.22 (0.05)
E. moraviensis	M2	0.61	−356			0.90 (1.07)	2.81 (0.90)	0.22 (0.07)
E. moraviensis	M3	0.04	−119			12.90 (17.75)	1.83 (0.14)	0.26 (0.005)
E. moraviensis	M4	0.01	−114	0.98 (0.15)	0.21 (0.01)		2.86 (2.86)	0.14 (0.14)

a The correlations of observed and fitted data are reflected by *R*^2^. The value in the parentheses is SD, which stands for parameter standard deviation.

In the validation step, we used the mean calibrated parameters from all columns in direct modeling to explain E. coli and E. moraviensis transport in columns 4 and 5 and column 5, respectively. According to the validation results (Fig. 4 and 6), the M1 model prediction is closer to measured data in explaining E. coli transport than other models, whereas the M2 model prediction is closer to observed data in explaining E. moraviensis transport. The large attachment coefficient for both bacteria highlights the relevance of bacteria attachment in the column, although the applied models are unable to discriminate between adsorption to the AWI and adsorption to the SWI. E. moraviensis showed a higher attachment rate coefficient (*K*_att_) than E. coli for a given model. Mass balance tables (Table 3 and Tables S1-S4 in the Supplemental materials) show a large rate of retention for E. moraviensis. This is in line with the estimated values of parameters from M1 given in Table 4, which shows that the attachment coefficient of E. moraviensis is larger than E. coli.

In this study, retained microorganisms in columns appeared in the effluent when rainfall events increase the water content in the column (Fig. 3 to 6). During imbibition, cells that were held at the AWI, film straining, or the air-water-solid triple point were released due to the dissolution of the AWI and the growth of water films (35, 36). During the entire wetting process, just a small percentage of bacteria was discharged from the soil column (Fig. 3 to 6). This might be since the saturation level at the air-water interface did not reach a critical level, which is related to capillary pressure, causing cells at the soil-water interface to remain linked (14). As a result of high surface tension, the soil-water interface has a higher impact on retaining E. coli during drainage and imbibition of the sand medium than the air-water interface (14). Furthermore, bacteria decay in the column and bacteria adhere to soil particles and surfaces. This is in line with the estimated value of attachment parameters and the relatively small number of bacteria in the outflow compared to the inflow. The trapped bacteria on the soil-water interface are subjected to various forces as the water film thickness grows during infiltration, which changes the hydrodynamic shear forces at a given point. This induces a percentage of attached cells to partition into the AWI at the wetting front, mobilizing them to the aqueous phase (14, 37). In this investigation, we assume that the above-described mechanism is the cause of bacteria release during irrigation events. (Fig. 3 to 6).

The attachment of bacteria to sand grains is largely dependent on the amount of water in a porous media (14). The water films around sand grains are thinner at low water content than at higher water content, reducing the distance between these grains and the bacteria and thus increasing the strength of the adhesive forces.

The results show that bacterium type affects transport and retention behaviors (Table 3). Larger bacteria are likely to have better retention and less movement. For example, E. moraviensis is known to cluster and form pairs (diplococci), short chains, or groups (38), which might result in greater sand retention by straining or bridging processes (10). This is also visible in our column experiments. Furthermore, Gram-negative bacteria were shown to attach to the sand more than Gram-positive bacteria (39). This may only be connected to the Gram stain type result by coincidences (39). Gram staining was developed to determine if a lipopolysaccharide outer membrane exists outside the cell wall (Gram positive) or not (Gram negative). It is uncertain how this outer component influences surface interactions (40). However, in this investigation, E. moraviensis, a Gram-positive bacteria, was attached to the sand more than E. coli, a Gram-negative bacteria. The cell wall composition, including surface charge, porous nature, and roughness, plays a crucial role in surface interactions and transport dynamics (40). Thus, the variations in cell wall composition, including surface charge and the porous nature of the cell surface, are believed to influence the attachment and transport behaviors of bacteria within porous media. We concluded that the size, shape, and cell properties, like the cell wall composition of the bacteria, as well as the properties of the porous media, are the dominant factors controlling bacteria transport.

### Bacterial retention.

**FIG 7:**
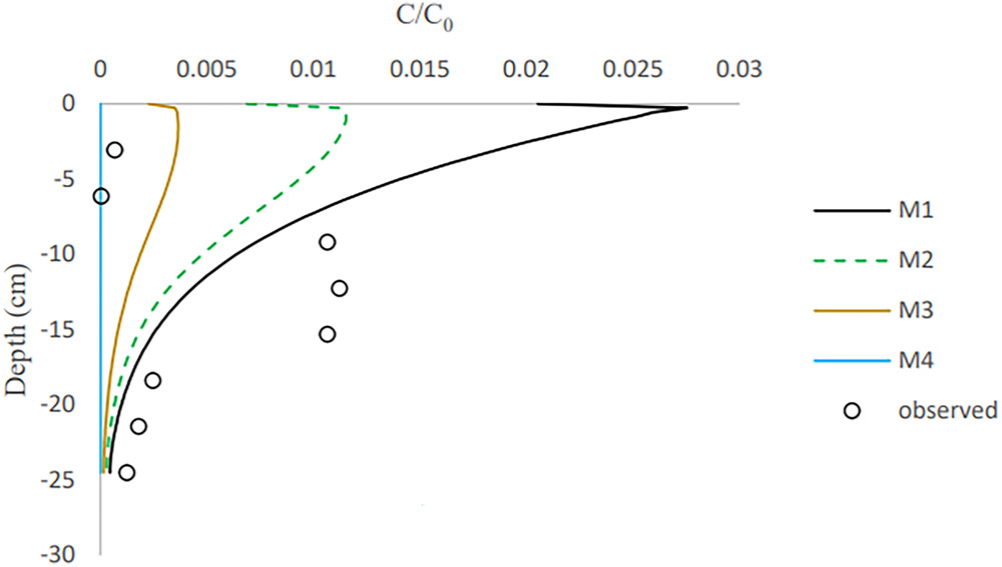
Predicted and measured profile retention for E. coli in column 1.

**FIG 8:**
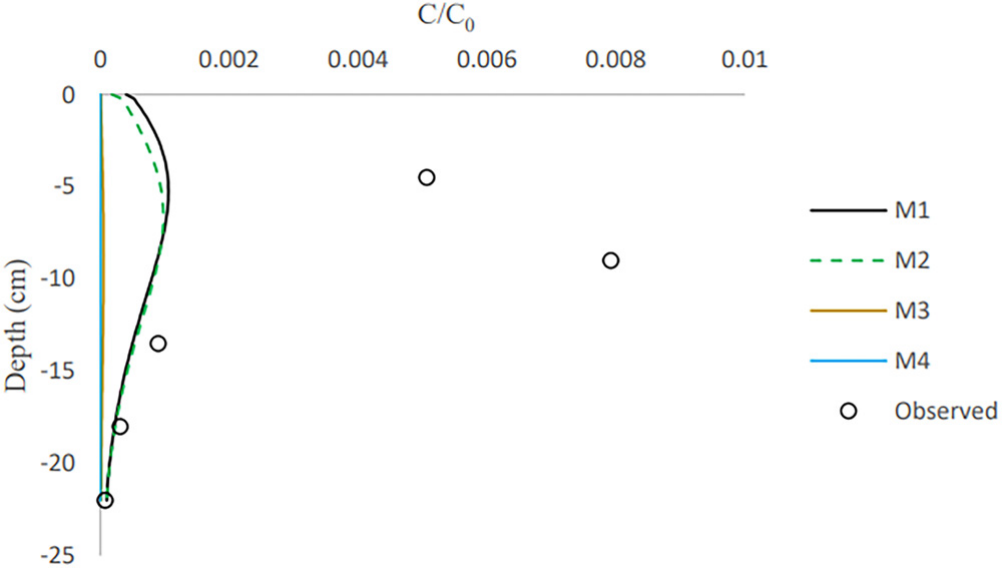
Predicted and measured profile retention for E. moraviensis in column 3.

Based on the final observed and simulated vertical distribution of retained bacteria in the columns (Fig. 7 and 8), E. coli retention increased until it reached its largest value at 10 to 15 cm depth in columns 1 and 2 and 9 cm depth in column 3 and then dropped as depth increased. In other words, detachment took place at the column’s top, and downward transport caused the spread of bacteria to deeper layers. E. moraviensis, on the other hand, retained highly at the column inlet, and subsequently, concentrations rapidly reduced at larger depths. For all models, there were differences between experimental and simulated retention patterns (Fig. 7). M1 and M2 had a fair description for bacteria retention at depths of 13.5 to 22 cm for E. moraviensis in column 3 (Fig. 8), and all of the models underestimate bacteria concentration in the sand in column 2 (see Fig. S8-S9 in the Supplemental materials). Also, other experimental studies have shown that the Langmuirian and attachment models may show deviation from the straining of colloids in the smallest region of the porous media (34, 41).

The measured profile retentions for both bacteria showed a rapid decline with distance. In the literature, similar distribution patterns with a steep reduction in concentration have been documented several times (33, 42, 43). Recognized reasons for this behavior include surface roughness, chemical heterogeneity, particle aggregation, and hydrodynamic variables (34, 44).

Many studies found that bacteria retention is strongly depth dependent (45–47). As previously noted, E. moraviensis can form clusters, resulting in straining and a greater pronounced rapid decline in concentration with distance than E. coli. Since bacteria have adhered to the sand grains, the concentration of the inoculation influent decreases as it is transported downward through the sand. The amount of bacteria available for adhesion to the lower regions of the column reduces as the downward-migrating pore water becomes depleted of bacteria. Furthermore, because of the above-mentioned influence of water content on bacterial attachment, attachment is more effective in the drier upper parts of the sand column than in the wet lower parts.

### Bacteria fate.

Bacteria from the soil surface can enter the porous media through irrigation or rainfall, where they can attach to the interfaces. Coefficients for bacterial attachment based on model calibration, such as physical adsorption at porous medium interfaces and detachment from interfaces, were provided through inverse simulations (Table 4). We found that the one-site model and Langmuirian model are capable of simulating the experimental result for E. coli. The Langmuirian model is capable of simulating the experimental result for E. moraviensis. Moreover, we were able to validate achieved coefficients with two columns in the case of E. coli and one column in the case of E. moraviensis. The results of bacteria transport simulated with the M1 model for E. coli and with M2 for E. moraviensis were in line with model-fitting results. Accumulated bacteria in porous media can detach and join the passing water if some transient water content occurs there due to irrigation or rainfall (Fig. 3 to 6). Then bacteria can accumulate in the porous medium’s deeper layers (Fig. 7 and 8), and eventually, they can leave the porous media and enter the groundwater (Fig. 3 to 6). Accumulated bacteria in the vadose zone can detach due to water content increase (Fig. 3 to 6), are transported to deeper layers (Fig 7 and 8), and eventually enter the saturated groundwater. According to our mass balance data and numerical modeling, the shape, size, and cell wall composition of the bacteria may affect retention mechanisms such as attachment (Tables 3 and 4). Other relevant factors are the characteristics of porous media, such as grain size and water content. In this research, the rate of retention varied significantly depending on the type of bacteria due to differences in size and shape of bacteria. Therefore, subsurface water pollution is more likely when a bacterial source, such as E. coli, is applied to sandy soil than when E. moraviensis is applied.

### Conclusion.

This study highlights the effects of transient water contents on the remobilization of fecal indicator bacteria in unsaturated porous media. After bacteria were inoculated into the sand columns, more than 99% were retained in the sand due to a combination of attachment and high decay rates. The measurements of the rainfall experiments revealed that with each succeeding rainfall event, the number of microorganisms discharged decreased. This is owing to the diminishing amount of bacteria in the soil at the start of each subsequent rainfall event, as well as the fact that the most accessible bacteria are released first, making it increasingly difficult to release bacteria with each rainfall event. E. moraviensis bacteria attach to sand grains more easily than E. coli bacteria and are more difficult to remobilize once attached. In the unsaturated zone, E. coli is relatively mobile and may move with percolating soil water to groundwater. The presented model output is useful in predicting bacterial contamination travel distance in porous media. This information can be used to determine safe locations of drinking water wells with respect to bacterial contamination. While caution must be exercised when extrapolating these findings to the field scale, they can serve as a useful cautionary indicator for understanding the potential risks associated with bacteria contamination in larger, real-world scenarios.

The retention profiles revealed that the majority of bacteria were retained at the porous medium's surface, particularly in the case of E. moraviensis. E. coli transport can be modeled using the one-site attachment/detachment model and the Langmuirian dynamic model, and E. moraviensis can be modeled using the Langmuirian dynamic model. Analysis of model parameters demonstrated that attachment and detachment are the most important mechanisms in E. coli and E. moraviensis transport in unsaturated porous media. However, attachment to two separate sites, such as SWI and AWI, could not be discriminated from each other based on the experimental and modeling results of this work.

The results of this study suggest that between microbial contamination at the soil surface and the groundwater, the unsaturated zone acts as an important barrier. Bacteria accumulating in the unsaturated zone, on the other hand, can accumulate to such amounts that they can be discharged into the saturated zone when saturation changes due to heavy rain events or groundwater level rise. In the case of E. coli, the risk of groundwater contamination exists. E. moraviensis is more difficult to remobilize once attached to porous media than E. coli. To properly differentiate the mechanisms governing bacterial remobilization, more studies and modeling are required. In general, by continuously monitoring bacteria concentration in the column effluent rather than in fractions, the experimental findings can be improved. As a result, the breakthrough curves will be smoother and have a greater resolution, which will enhance parameter calibration. In addition, more research is required to apply the results to other soil types and other microorganisms as well as cotransport of these bacteria.

## MATERIALS AND METHODS

### Soil column.

**FIG 9:**
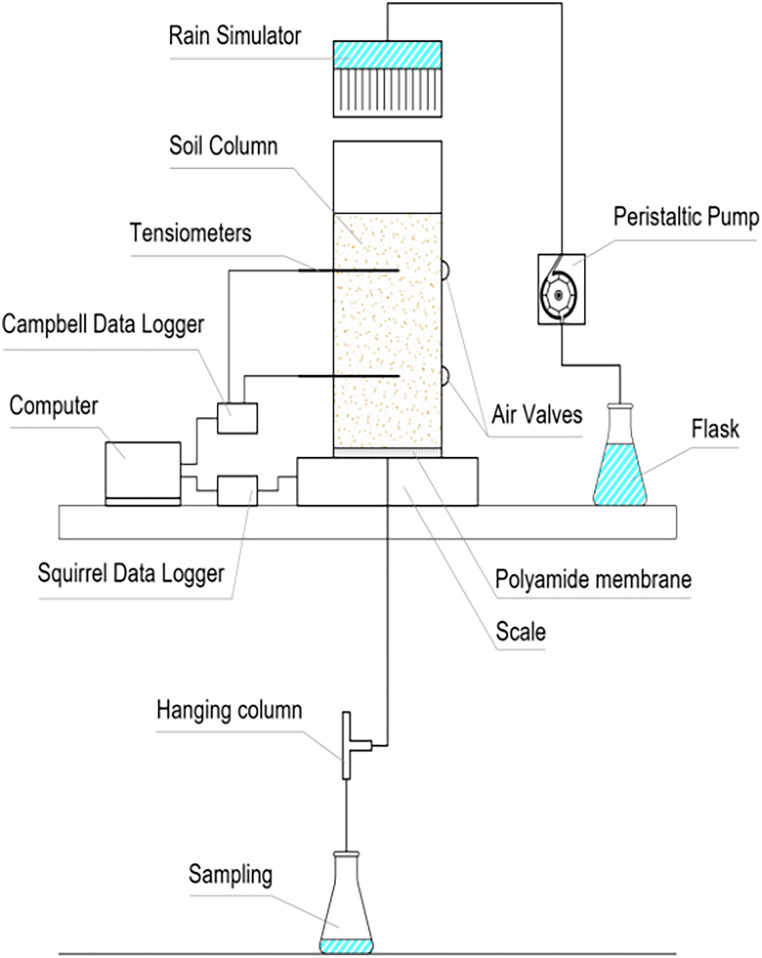
Scheme of the experimental setup used in the tracer and bacteria experiment.

Sand samples with a median grain size (*d*_50_) of 325 μm were collected at the aquifer recharge site in the Castricum dune area (52.538793°E and 4.617135°N) from a depth of 50 to 70 cm. The sand was used to pack five transparent polyvinyl chloride (PVC) columns with a height of 30 cm and an interior diameter of 15.2 cm. The moist sand was uniformly packed in layers of about 2.5 cm until it reached a height of 24.5 cm in two of the columns and 22 cm in three others, with a bulk density of 1.65 g/cm^3^. A rain simulator with hypodermic needles attached to a peristaltic pump (Barnant Company, Barrington, IL, USA) conveyed the tracer solution and bacteria suspension to the top of the column. Two autofill tensiometers (Rhizosphere Research Product, Wageningen, the Netherlands) were implanted at 6 and 17 cm from the top of the column to measure the soil water pressure, and a Campbell data logger (Campbell Scientific, Logan, UT) was used to capture their data. To enable air entry and release during the imbibition and drainage processes, air valves were placed on the wall opposite the tensiometers. At the bottom of the column, a polyamide woven filter cloth (Sefar) with a nominal pore size of 10 μm was installed, which prevents sand from getting through but allows bacteria to freely travel through its pores. Based on nominal pore size, the air entry value was estimated to be circa −100 cm. It is possible that the filter cloth could serve as an additional barrier for bacteria; however, we did not specifically investigate the extent of bacterial attachment to the filter cloth in our study. In future studies, it would be valuable to investigate the bacterial attachment to the filter cloth and evaluate its influence on overall transport processes within unsaturated porous media. To create a fixed unsaturated condition at the column bottom, the membrane was connected to a pressure head of −30 cm by a hanging water column (Fig. 9). During the experiment, the columns were placed on scales to measure their change in water content. Effluent samples from the hanging column were collected and analyzed for tracer and bacteria concentrations. To mimic soil temperatures, the columns were placed in a conditioned room at 15°C.

### Bacteria.

The bacteria E. coli and E. moraviensis were used in the experiment, as they are fecal indicator bacteria utilized in the analysis of drinking water quality. The bacterial strains employed in this investigation were isolated from water sampled at the infiltration area of the Castricum dunes (the Netherlands). E. coli is a Gram-negative rod-shaped cell that produces minimal amounts of lipopolysaccharides and extracellular polymeric substances (14). E. moraviensis is a Gram-positive ovoid cell occurring as a single cell, in pairs, short chains, or in small groups, elongated in the direction of chains, nonpigmented, and nonmotile (38). Before beginning the experiment, the sand was examined to determine the initial concentration of E. coli and E. moraviensis, and the sand did not contain these specific bacteria.

In order to prepare the bacterial suspension, pure cultures of E. coli and E. moraviensis were utilized, having been previously isolated and characterized. Both strains were subjected to incubation in Lab-Lemco powder (8 g/L). The bacterial cultures were maintained under aerobic conditions at their respective optimal growth temperatures of 36°C ± 2°C for 48 h, ensuring the bacteria reached the exponential-growth phase and were in an actively metabolizing state.

Following the incubation period, the bacterial cultures underwent centrifugation at 3,000 × *g* for 20 min to collect the cellular pellets. No brake was applied to decelerate the centrifuge after the 20-min duration. Subsequently, the supernatant was cautiously discarded, and the bacterial pellets were resuspended in sterile phosphate-buffered saline (PBS), resulting in a concentrated bacterial suspension. This resuspension process was repeated 2 to 3 times to ensure thorough removal of any residual nutrients.

By employing these steps, a concentrated bacterial suspension was successfully prepared, utilizing previously isolated and characterized pure cultures of E. coli and E. moraviensis. The incubation under optimal conditions followed by centrifugation and resuspension in sterile PBS ensured the availability of actively metabolizing bacteria for subsequent experimental procedures while minimizing the presence of extraneous nutrients.

### Transport experiments.

In order to derive soil hydraulic properties, a salt tracer experiment was carried out before bacteria were applied to the unsaturated soil columns. A 0.58-mM NaCl solution was supplied to the top of each of the five unsaturated columns at a rate of 12.5 mm/h for 4 h by a rainfall simulator. The electrical conductivity of effluent was measured per minute at the outflow of the hanging column using an electrical conductivity probe. After 24 h, the columns were flushed with a 1.5 pore volume of tap water for 4 h to remove salt from the columns.

Next, a rainfall simulator was used to apply a 1.5 pore volume of bacteria suspension containing both E. coli and E. moraviensis to each sand column at a rate of 12.5 mm/h for 4 h. After 24 h from the start of the experiment, the column leachate was collected in a single sample for bacterial analysis (E. coli and E. moraviensis). Because the volume and concentration of bacteria in the influent and effluent are known, the absolute number of bacteria retained in the soil column after inoculation can be calculated. The total number of retained bacteria for rain event experiments was assessed at this step.

Following that, three 4-h rainfall events with tap water and a 12.5-mm/h intensity were carried out for all five columns, separated by 24 h. The effluent from each rainfall event was collected in 100-mL samples, giving a total of 9 effluent samples to analyze the concentration of bacteria transported with water. The average concentration of bacteria was determined by microbial analysis of the samples. The total number of bacteria released per rainfall was calculated from the collected effluent fractions.

To determine the final distribution of bacteria in the soil at different depths, sand samples were taken from all five columns using a hollow metal rod with an inner diameter of 5 cm at the end of the experiment. This sampling method was utilized to extract a small soil profile from the column.

### Bacterial analysis.

The bacteria concentrations in influent and effluent samples were determined at the undiluted sample and four dilutions, 1:10, 1:100, 1:1,000, and 1:10,000, in order to obtain countable results. For E. coli and E. moraviensis, 0.1 mL of each dilution, as well as the undiluted sample, was plated on a petri dish containing lauryl sulfate agar (LSA) and Slanetz and Bartley medium (S&B medium), respectively. Each undiluted sample was also filtered through a cellulose nitrate (CN) membrane filter with a pore size of 0.45 μm and plated to detect very low concentrations of E. coli or E. moraviensis, respectively. After that, the dishes for E. coli screening were incubated for 5 h at 25°C, followed by 14 h at 36°C. The plates were inoculated at 36°C for 44 h to determine E. moraviensis. The number of bacteria was set equal to the number of CFU after incubation. The concentrations of both bacteria in the influent suspensions were determined using the method described above before each experiment.

### Bacteria decay.

The decay rate of E. coli and E. moraviensis bacteria was determined under the same conditions as the column experiments for 7 days. For this, 80 g of sand and bacteria suspension were added to conical tubes, and the tubes were covered with a lid that allowed air to pass through but limited evaporation. During the experiment period, two tubes were collected every day to determine the bacteria concentration. Then, the sand from the tubes was mixed with 500 mL sterile tap water and shaken for 2 min, followed by a 2-min rest period to allow the sand to settle. The liquid above the settled sand was collected, and the number of E. coli and E. moraviensis in this volume of liquid was determined.

The decay rate of fecal bacteria is usually described by a first-order process with the following equation:

(1) $$\frac{C_{t}}{C_{0}}\;=e^{-\text{μ}t}$$

where *C*_0_ and *C_t_* are the concentrations of microorganisms at time *t*_0_, and *t* (*T*) and μ is the first-order decay constant (*T*^−1^) (48).

### Modeling and data analysis.

To determine the soil moisture characteristic of the sand samples, a sandbox (49) and a pressure plate were used. Using these moisture characteristic data, the van Genuchten model parameters (50) α, *n*, and ϴ*_r_* were fitted. These soil hydraulic parameters are related to the inverse of the air entry value, the width of the pore size distribution, and residual water content, respectively. Data from the NaCl breakthrough curves were then used to optimize the saturated hydraulic conductivity, *K_s_*; saturated water content, ϴ*_s_*; hydraulic tortuosity factor, *l*; and dispersivity, λ, using the inverse simulation mode of HYDRUS-1D (51). Subsequently, the optimized soil hydraulic parameters and optimized dispersivity were used in the inverse simulation runs to calibrate bacteria transport and retention parameters during the column rainfall experiments.

The concentration of bacteria in effluent samples during inoculation and rainfall events, as well as the concentration of bacteria in sand samples at the end of the experiments in columns 1, 2, and 3, was used to calibrate E. coli transport and retention. We used the same set of parameters for validation (direct modeling) to explain E. coli transport for columns 4 and 5. For E. moraviensis transport inverse modeling, data from columns 1 and 2 could not be used, as the outflow was too small. Therefore, in the analysis of E. moraviensis transport, data from columns 3 and 4 were utilized for calibration, and data from column 5 were used for validation.

The vadose zone model HYDRUS-1D was used to analyze the transport experiments (51). HYDRUS-1D offers different concepts to describe bacteria transport. Equations 2 and 3 are the governing equations for the two-kinetic site model.

(2) $$\text{θ}\frac{\partial C}{\partial t}+\rho_{b}\frac{\partial S_{1}}{\partial t}+\rho_{b}\frac{\partial S_{2}}{\partial t}=\lambda \theta v\frac{\partial^{2}C}{\partial x^{2}}-\theta v\frac{\partial C}{\partial x}-\mu_{w}\theta C-\mu_{s1}\rho_{b}S_{1}-\mu_{s2}\rho_{b}S_{2}$$

where θ is the volumetric water content, *C* is the bacterial concentration in the aqueous phase *N_c_L*^−3^ (*N_c_* is the number of colloids), *S* is the concentration of attached bacteria (*N_c_M*^−1^), ρ*_b_* is bulk density (ML^−3^), *x* is the distance in the vertical direction *L*, *t* is time *T*, μ*_w_* and μ*_s_* represent growth/decay of the free and attached bacteria (*T*^−1^), and the subscripts 1 and 2 refer to the two different sorption sites (42, 52). ∂ is partial derivative. It signifies the rate of change of a dependent variable with respect to an independent variable, indicating how a specific parameter varies as another factor is modified. The mass transfer between the soil solution and the *S*_1_ and *S*_2_ sites (52) is described as follows:

(3) $$\rho_{b}\frac{\partial S}{\partial t}=\rho_{b}\frac{\partial (S_{1}+S_{2})}{\partial t}=\theta \psi K_{\text{att1}}C-\rho_{b}K_{\text{det1}}S_{1}+\theta \psi K_{\text{att2}}C-\rho_{b}K_{\text{det2}}S_{2}$$

where *K*_att_ and *K*_det_ are the attachment and detachment rate coefficients, respectively, and (*T*^−1^), ψ(−) is a dimensionless function to account for time and depth-dependent retention. The subscripts 1 and 2 refer to the two different kinetic sites. Equations 2 and 3 are written for two kinetic sites, but by setting the attachment and detachment coefficients of the second site to zero, they can be applied to a single kinetic site. Bradford et al. (34) proposed a flexible function to account for time and depth-dependent deposition behavior, which is as follows:

(4) $$\psi =\left(1-\frac{S}{S_{\text{max}}}\right){\left(\frac{d_{c}+z}{d_{c}}\right)}^{-\beta }$$

where $S_{\text{max}}$ is the maximum solid-phase concentration (*N_c_ M*^−1^) of colloids on sorption sites, $d_{c}$ represents the mean diameter of the sand particle *L*, *z* represents the depth where the straining process begins, and β is an empirical factor controlling the shape of the spatial distribution (−). Bradford et al. (34) discovered that using the value of β of 0.432 in the simulations provided the best simulation of studies with significant depth-dependent deposition (34).

Four model formulations based on equations 2 to 4 are investigated in this work. By setting ψ to 1 and setting the second sorption site equal to zero, model M1 refers to a one-site attachment/detachment model. The Langmuirian dynamic (ψ < 1) is used to describe attachment and detachment in model M2. By setting β to 0, model M3 refers to attachment/detachment with Langmuirian and blocking of favorable deposition sites. The two-site attachment/detachment model and model fitted based on equations 2 to 4 is referred to as model M4.
