# Supplementary material for: Transport and Retention of Fecal Indicator Bacteria in Unsaturated Porous Media: Effect of Transient Water Flow
Source: Appl Environ Microbiol. 2023 Jul 17;89(8):e00219-23. doi: 10.1128/aem.00219-23 (PMC10467344; doi:10.1128/aem.00219-23)
Supplement: Supplemental file 1 — Supplemental material. Download aem.00219-23-s0001.docx, DOCX file, 0.6 MB [file aem.00219-23-s0001.docx]

**Transport and retention of fecal indicator bacteria in unsaturated porous media: effect of transient water flow**

Rozita Soltani Tehrani^a*^, Luc Hornstra^b^, Jos van Dam^a^, Gijsbert Cirkel^b^

^a^ Department of Soil Physics and Land Management, Wageningen University and Research, P.O. Box 47, 6700 AA Wageningen, the Netherlands
^b^ KWR Water research Institute, Nieuwegein, the Netherlands

Email addresses: [rozita.soltanitehrani@wur.nl](mailto:rozita.soltanitehrani@wur.nl) (R. Soltani Tehrani), luc.hornstra@tno.nl (L. Hornstra), [jos.vandam@wur.nl](mailto:jos.vandam@wur.nl) (J. van Dam) [Gijsbert.Cirkel@kwrwater.nl](mailto:Gijsbert.Cirkel@kwrwater.nl) (G. Cirkel)

Fit the tracer breakthrough curve with the advection-dispersion equation to estimate the saturated hydraulic conductivity *Ks*, saturated water content *ϴs*, hydraulic tortuosity factor *l*, and dispersivity *λ* of the porous medium

Number of calibrated parameters = 4 (saturated hydraulic conductivity, saturated water content, hydraulic tortuosity factor, and dispersivity)

Fit the breakthrough curve of *E. moraviensis* in columns 3 and 4 with M1(one-site attachment-detachment), M2 (Langmuirian), M3 (Langmuirian with blocking), and M4 (Two-site depth dependent) models to estimate the values of parameters describing the behavior of E. moraviensis in the porous media.

Number of calibrated parameters in M1 = 2 (Katt_1_, Kdet_1_)

Number of calibrated parameters in M2 = 3 (Katt_1_, Kdet_1_, Smax1)

Number of calibrated parameters in M3 = 3 (Katt_1_, Kdet_1_, Smax1)

Number of calibrated parameters in M4 = 4 (Katt_1_, Kdet_1_, Katt_2_, Kdet_2_)

Fit the breakthrough curve of *E. coli* in columns 1, 2, and 3 with M1(one-site attachment-detachment), M2 (Langmuirian), M3 (Langmuirian with blocking), and M4 (Two-site depth dependent) models to estimate the values of parameters describing the behavior of *E. coli* in the porous media.

Number of calibrated parameters in M1 = 2 (Katt_1_, Kdet_1_)

Number of calibrated parameters in M2 = 3 (Katt_1_, Kdet_1_, Smax1)

Number of calibrated parameters in M3 = 3 (Katt_1_, Kdet_1_, Smax1)

Number of calibrated parameters in M4 = 4 (Katt_1_, Kdet_1_, Katt_2_, Kdet_2_)

Validate average values of calibrated parameters for M1, M2, M3, and M4 in column 5 to predict *E. moraviensis* transport.

Validate average values of calibrated parameters for M1, M2, M3, and M4 in columns 4 and 5 to predict *E. coli* transport.

FIG S1. Modeling procedure for *E. coli* and *E. moraviensis* transport in soil columns

FIG S2. Measured and fitted bacterial die-off curve for *E. coli*

FIG S3. Measured and fitted bacteria die-off curve for *E. moraviensis*

TABLE S1. Experimental mass balance of *E. coli* and *E. moraviensis* for column 1

| Mass type | Bacteria | | | |
| --- | --- | --- | --- | --- |
|  | *E. coli* | | *E. moraviensis* | |
|  | CFU | Percentage | CFU | Percentage |
| Influent | 2.28E+10 | 100 | 1.37E+08 | 100 |
| Effluent | 1.48E+06 | 0.006 | 2.53E+04 | 0.01853 |
| First-rain | 2.98E+05 | 0.00131 | 3.39E+02 | 0.00025 |
| Second-rain | 1.76E+05 | 0.00077 | 1.77E+02 | 0.00013 |
| Third-rain | 9.71E+04 | 0.0004 | 9.13E+01 | 0.00007 |
| Remain in the column | 1.03E+09 | 4.53 | 1.19E+08 | 87 |
| Decay rate | 1.98E+10 | 87 | 6.19E+07 | 45 |
| Error | 1.96E+09 | 9 | -4.39E+07 | -32 |

TABLE S2. Experimental mass balance of *E. coli* and *E. moraviensis* for column 3

| Mass type | Bacteria | | | |
| --- | --- | --- | --- | --- |
|  | *E. coli* | | *E. moraviensis* | |
|  | CFU | Percentage | CFU | Percentage |
| Influent | 1.01E+11 | 100 | 8.07E+09 | 100 |
| Effluent | 5.15E+07 | 0.051 | 9.06E+04 | 0.001 |
| First-rain | 7.00E+06 | 0.007 | 1.86E+05 | 0.002 |
| Second-rain | 2.95E+06 | 0.003 | 1.05E+05 | 0.001 |
| Third-rain | 7.01E+05 | 0.001 | 2.88E+04 | 0.0004 |
| Remain in the column | 1.26E+10 | 12 | 2.36^E^+09 | 29 |
| Decay rate | 8.80E+10 | 87 | 3.65E+09 | 45 |
| Error | 3.79^E^+08 | 0.38 | 2.06E+09 | 25 |

TABLE S3. Experimental mass balance of *E. coli* and *E. moraviensis* for column 4

| Mass type | Bacteria | | | |
| --- | --- | --- | --- | --- |
|  | *E. coli* | | *E. moraviensis* | |
|  | CFU | Percentage | CFU | Percentage |
| Influent | 1.00E+11 | 100 | 9.42E+08 | 100 |
| Effluent | 3.74E+06 | 0.004 | 1.56E+02 | 0.00002 |
| First-rain | 2.62E+05 | 0.0003 | 3.50E+03 | 0.0004 |
| Second-rain | 6.81E+05 | 0.0007 | 1.17E+03 | 0.0001 |
| Third-rain | 4.38E+05 | 0.0004 | 7.88E+02 | 0.00008 |
| Remain in the column | 1.24E+10 | 12 | 2.54E+08 | 27 |
| Decay rate | 8.699E+10 | 87 | 4.26E+08 | 45 |
| Error | 6.05E+08 | 0.60 | 2.62E+08 | 28 |

TABLE S4. Experimental mass balance of *E. coli* and *E. moraviensis* for column 5

| Mass type | Bacteria | | | |
| --- | --- | --- | --- | --- |
|  | *E. coli* | | *E. moraviensis* | |
|  | CFU | Percentage | CFU | Percentage |
| Influent | 5.98E+10 | 100 | 1.08E+09 | 100 |
| Effluent | 1.51E+06 | 0.002 | 1.59E+02 | 0.00001 |
| First-rain | 1.27E+05 | 0.0002 | 7.01E+02 | 0.00006 |
| Second-rain | 1.15E+05 | 0.0002 | 2.70E+02 | 0.00002 |
| Third-rain | 1.37E+05 | 0.0002 | 8.08E+01 | 0.000007 |
| Remain in the column | 1.24E+10 | 21 | 2.54E+08 | 23.54 |
| Decay rate | 5.20E+10 | 87 | 4.87E+08 | 45 |
| Error | -4.56E+09 | -8 | 3.39E+08 | 31 |

FIG S4. Calibration: Observed and simulated breakthrough curves for *E. coli* in Column experiment 1

FIG S5. Calibration: Observed and simulated breakthrough curves for *E. coli* in Column experiment 3

FIG S6. Validation: Observed and simulated breakthrough curves for *E. coli* in Column experiment 4

FIG S7. Calibration: Observed and simulated breakthrough curves for *E. moraviensis* in Column experiment 3

FIG S8. Predicted and measured profile retention for *E. coli* in column 2

FIG S9. Predicted and measured profile retention for *E. coli* in column 3

FIG S10. Predicted and measured profile retention for *E. moraviensis* in column 4
